# Supplementary material for: Unveiling the Bioactive Potential of the Invasive Jellyfish Phyllorhiza punctata Through Integrative Transcriptomic and Proteomic Analyses
Source: Biomolecules. 2025 Aug 4;15(8):1121. doi: 10.3390/biom15081121 (PMC12383608; doi:10.3390/biom15081121)
Supplement: Supplementary file 1 [file biomolecules-15-01121-s001.zip › Suplementary Material/FileS3/PEPFOLD4/XP_065054305_554/clusters.html]

 PEP-FOLD clusters


Download archive of all models  here   

|  |  |  |
| --- | --- | --- |
| # | file | sOPEP |
| # cluster 1 - Size: 8 |  |  |
|  | XP\_065054305\_554-model1 | -21.1739 |
|  | XP\_065054305\_554-model1.1 | -21.0412 |
|  | XP\_065054305\_554-model1.2 | -20.7239 |
|  | XP\_065054305\_554-model1.3 | -20.7204 |
|  | XP\_065054305\_554-model1.4 | -20.7141 |
|  | XP\_065054305\_554-model1.5 | -20.6836 |
|  | XP\_065054305\_554-model1.6 | -20.6836 |
|  | XP\_065054305\_554-model1.7 | -20.6706 |
| # cluster 2 - Size: 11 |  |  |
|  | XP\_065054305\_554-model2 | -21.0787 |
|  | XP\_065054305\_554-model2.1 | -20.9812 |
|  | XP\_065054305\_554-model2.2 | -20.9167 |
|  | XP\_065054305\_554-model2.3 | -20.9032 |
|  | XP\_065054305\_554-model2.4 | -20.8464 |
|  | XP\_065054305\_554-model2.5 | -20.8286 |
|  | XP\_065054305\_554-model2.6 | -20.7767 |
|  | XP\_065054305\_554-model2.7 | -20.7323 |
|  | XP\_065054305\_554-model2.8 | -20.6737 |
|  | XP\_065054305\_554-model2.9 | -20.659 |
|  | XP\_065054305\_554-model2.10 | -20.6428 |
| # cluster 3 - Size: 41 |  |  |
|  | XP\_065054305\_554-model3 | -21.0247 |
|  | XP\_065054305\_554-model3.1 | -21.0199 |
|  | XP\_065054305\_554-model3.2 | -20.9934 |
|  | XP\_065054305\_554-model3.3 | -20.9237 |
|  | XP\_065054305\_554-model3.4 | -20.8876 |
|  | XP\_065054305\_554-model3.5 | -20.8876 |
|  | XP\_065054305\_554-model3.6 | -20.8876 |
|  | XP\_065054305\_554-model3.7 | -20.8768 |
|  | XP\_065054305\_554-model3.8 | -20.868 |
|  | XP\_065054305\_554-model3.9 | -20.8639 |
|  | XP\_065054305\_554-model3.10 | -20.8639 |
|  | XP\_065054305\_554-model3.11 | -20.8596 |
|  | XP\_065054305\_554-model3.12 | -20.8596 |
|  | XP\_065054305\_554-model3.13 | -20.8596 |
|  | XP\_065054305\_554-model3.14 | -20.8499 |
|  | XP\_065054305\_554-model3.15 | -20.8499 |
|  | XP\_065054305\_554-model3.16 | -20.8419 |
|  | XP\_065054305\_554-model3.17 | -20.8419 |
|  | XP\_065054305\_554-model3.18 | -20.8419 |
|  | XP\_065054305\_554-model3.19 | -20.8259 |
|  | XP\_065054305\_554-model3.20 | -20.822 |
|  | XP\_065054305\_554-model3.21 | -20.8217 |
|  | XP\_065054305\_554-model3.22 | -20.8217 |
|  | XP\_065054305\_554-model3.23 | -20.8217 |
|  | XP\_065054305\_554-model3.24 | -20.8217 |
|  | XP\_065054305\_554-model3.25 | -20.8217 |
|  | XP\_065054305\_554-model3.26 | -20.8156 |
|  | XP\_065054305\_554-model3.27 | -20.8036 |
|  | XP\_065054305\_554-model3.28 | -20.8011 |
|  | XP\_065054305\_554-model3.29 | -20.7569 |
|  | XP\_065054305\_554-model3.30 | -20.7402 |
|  | XP\_065054305\_554-model3.31 | -20.7301 |
|  | XP\_065054305\_554-model3.32 | -20.7282 |
|  | XP\_065054305\_554-model3.33 | -20.6971 |
|  | XP\_065054305\_554-model3.34 | -20.6802 |
|  | XP\_065054305\_554-model3.35 | -20.6713 |
|  | XP\_065054305\_554-model3.36 | -20.649 |
|  | XP\_065054305\_554-model3.37 | -20.6485 |
|  | XP\_065054305\_554-model3.38 | -20.6347 |
|  | XP\_065054305\_554-model3.39 | -20.6039 |
|  | XP\_065054305\_554-model3.40 | -20.5987 |
| # cluster 4 - Size: 17 |  |  |
|  | XP\_065054305\_554-model4 | -20.9935 |
|  | XP\_065054305\_554-model4.1 | -20.9651 |
|  | XP\_065054305\_554-model4.2 | -20.9319 |
|  | XP\_065054305\_554-model4.3 | -20.8975 |
|  | XP\_065054305\_554-model4.4 | -20.8491 |
|  | XP\_065054305\_554-model4.5 | -20.7874 |
|  | XP\_065054305\_554-model4.6 | -20.7861 |
|  | XP\_065054305\_554-model4.7 | -20.775 |
|  | XP\_065054305\_554-model4.8 | -20.7684 |
|  | XP\_065054305\_554-model4.9 | -20.7498 |
|  | XP\_065054305\_554-model4.10 | -20.74 |
|  | XP\_065054305\_554-model4.11 | -20.7062 |
|  | XP\_065054305\_554-model4.12 | -20.6839 |
|  | XP\_065054305\_554-model4.13 | -20.671 |
|  | XP\_065054305\_554-model4.14 | -20.5811 |
|  | XP\_065054305\_554-model4.15 | -20.5664 |
|  | XP\_065054305\_554-model4.16 | -20.5414 |
| # cluster 5 - Size: 9 |  |  |
|  | XP\_065054305\_554-model5 | -20.9926 |
|  | XP\_065054305\_554-model5.1 | -20.921 |
|  | XP\_065054305\_554-model5.2 | -20.8751 |
|  | XP\_065054305\_554-model5.3 | -20.8181 |
|  | XP\_065054305\_554-model5.4 | -20.8092 |
|  | XP\_065054305\_554-model5.5 | -20.7281 |
|  | XP\_065054305\_554-model5.6 | -20.6977 |
|  | XP\_065054305\_554-model5.7 | -20.6447 |
|  | XP\_065054305\_554-model5.8 | -20.6142 |
| # cluster 6 - Size: 13 |  |  |
|  | XP\_065054305\_554-model6 | -20.8836 |
|  | XP\_065054305\_554-model6.1 | -20.8515 |
|  | XP\_065054305\_554-model6.2 | -20.8487 |
|  | XP\_065054305\_554-model6.3 | -20.8373 |
|  | XP\_065054305\_554-model6.4 | -20.8265 |
|  | XP\_065054305\_554-model6.5 | -20.7944 |
|  | XP\_065054305\_554-model6.6 | -20.7908 |
|  | XP\_065054305\_554-model6.7 | -20.7886 |
|  | XP\_065054305\_554-model6.8 | -20.7775 |
|  | XP\_065054305\_554-model6.9 | -20.722 |
|  | XP\_065054305\_554-model6.10 | -20.7132 |
|  | XP\_065054305\_554-model6.11 | -20.7076 |
|  | XP\_065054305\_554-model6.12 | -20.6534 |
| # cluster 7 - Size: 1 |  |  |
|  | XP\_065054305\_554-model7 | -20.6266 |
|  |  |  |
